# Infant feeding guideline awareness among mothers living with HIV in North America and Nigeria

**DOI:** 10.1186/s13006-020-00274-z

**Published:** 2020-04-17

**Authors:** J. Craig Phillips, Josephine Etowa, Jean Hannan, Egbe B. Etowa, Seye Babatunde

**Affiliations:** 1 Faculty of Health Sciences, School of Nursing, University of Ottawa, Ottawa, Ontario Canada; 2 Nicole Wertheim College of Nursing and Health Sciences, Florida International University, Miami, Florida, USA; 3 Centre for Health and Development, University of Port Harcourt, Port Harcourt, Rivers State Nigeria

**Keywords:** Black mothers, Infant feeding guidelines, Awareness of guidelines, Exclusive breastfeeding, Exclusive formula feeding, HIV, HIV criminalization, HIV-positive mother, Maternal-child health, Motherhood

## Abstract

**Background:**

WHO guidelines recommend breastfeeding for mothers living with HIV adherent to antiretroviral therapy in countries where formula is not accessible. In Canada and the US, guidelines for mothers living with HIV recommend exclusive formula feeding. Awareness of national infant feeding guidelines and socio-cultural factors influence infant feeding choices that may result in an increased risk of vertical transmission of HIV. The purpose of this paper is to present factors associated with awareness of guidelines among Black mothers living with HIV. Data were derived from a survey conducted as part of a recent international study that examined infant feeding practices among Black women living with HIV in Ottawa, Canada; Port Harcourt, Nigeria; and Miami, Florida.

**Methods:**

Participants (*n* = 690) from Port Harcourt (*n* = 400), Miami (*n* = 201), and Ottawa (*n* = 89) were surveyed on their awareness of infant feeding guidelines for mothers living with HIV. Data were collected between November, 2016 and March, 2018.

**Results:**

Participants’ mean ages were 34.3 ± 5.9 years. Across all sites, 15.4% (95% CI 13.2, 7.7) of mothers were **NOT** aware of their country’s infant feeding guidelines. Cultural beliefs (OR = 0.133, *p* = 0.004, 95% CI 0.03, 0.53) and functional social support influenced infant feeding choices (OR = 1.1, *p* = 0.034, 95% CI 1.01, 1.20) and were statistically significant predictors of guideline awareness (*Χ*^*2*^ = 38.872, *p* < .05) after controlling for age, years of formal education, marital status, and country of residence. As agents of functional social support, family members and health workers (e.g., nurses, physicians, social workers, other health care workers) influenced participants’ awareness of infant feeding guidelines and guided them in their infant feeding choices.

**Conclusions:**

Among participants, awareness of national infant feeding guidelines was associated with functional social support and cultural beliefs influenced infant feeding choices. Therefore, culturally adapted messaging via social supports already identified by mothers, including family relationships and health workers, is an appropriate way to enhance awareness of infant feeding guidelines. Ultimately, contributing to the global health goals of maternal health and reduced infant mortality.

## Background

World Health Organization (WHO) guidelines recommend exclusive breastfeeding (EBF) for mothers living with HIV who are adherent with antiretroviral therapy (ART) in countries where formula is not accessible [1, 2]. This recommendation is premised on the notion that there may be a low risk of vertical transmission of HIV from a mother whose HIV viral load is undetectable [3]. In Canada and the US, guidelines limit mothers living with HIV to exclusive formula feeding (EFF) and in some jurisdictions those mothers that do not adhere to the guidelines could be prosecuted or lose custody of their infant to agents of the state (e.g., child protective services, police) for breastfeeding [4, 5].

Of utmost importance to successful implementation of infant feeding guidelines is creating awareness of the recommendations among health workers (e.g., nurses, physicians, social workers, other health care workers) and persons for whom the guideline was developed. Health workers may have knowledge deficits related to the infant feeding guidelines for HIV-infected mothers that include: lacking awareness or knowledge of the existence of these guidelines or they may lack understanding of the rationale or intent of the guidelines to prevent vertical HIV transmission from mother to infant. Increased awareness of safe infant feeding practices in the context of HIV infection has been associated with guideline adherence [6, 7]. All expectant mothers and their families need to be informed of the existence of the guidelines for mothers living with HIV.

Additionally, health workers may fail to communicate correct information or may not communicate at all about infant feeding options for mothers living with HIV. Health workers need adequate knowledge of guideline recommendations to effectively transfer that knowledge to their patients. Health workers who do not understand the rationale or intent of infant feeding guidelines may jeopardize the level of awareness they create for mothers living with HIV. In the US, 29% of mothers living with HIV breastfed despite health workers’ recommendations against breastfeeding [8]. African American women are less likely than other ethno-racial groups to adhere to policies advocating breastfeeding [9–11]. Intersecting sociocultural factors among African American women that influence adherence to these policies include: lack of parental knowledge regarding breastfeeding practices and lack of maternal access to information that promotes and supports breastfeeding [9–11]. These factors make it clear that creating awareness of infant feeding guidelines by health workers should be stepped up, for mothers with HIV as well as for the general population.

In addition to the influence of health workers, several other factors influence awareness of health actions such as national infant feeding guidelines for HIV positive mothers. Traditionally, the place of socio-demographic characteristics (e.g., age, formal education, occupation) as drivers of awareness or knowledge of health actions is well documented [8, 12]. Access to traditional sources of health information such as health workers or contemporary information media, such as the Internet, contribute to awareness of health promoting actions [8, 12].

In this study, we examined the influence of socio-cultural context and HIV-related constructs on awareness of infant feeding guidelines among Black mothers living with HIV in three cosmopolitan cities in Africa (Port Harcourt, Nigeria) and North America (Miami and Ottawa). This understanding of the experiences of indigenous Black women in Nigeria may inform promising solutions to enhance guideline awareness in Canada and the US. Likewise, Nigerian women may benefit from study findings from Canada and the US through opportunities for reciprocal knowledge sharing and exchange between all three national contexts. The socio-cultural perspective of the study necessitates this comparison to highlight how changes in cultural values due to migrations may influence awareness of safe infant feeding practices among mothers living with HIV.

## Methods

This study is part of a mixed methods community based participatory research project to examine the specific sub-culture of infant feeding while living with HIV in Port Harcourt, Nigeria; Ottawa, Canada; and Miami, Florida. The quantitative component of that research project included the current study, a cross-sectional multi-country survey to describe the socio-cultural and psychosocial factors that influenced infant feeding practices (i.e. EBF, mixed feeding, EFF) among these mothers. The current study described participants’ awareness of infant feeding guidelines for mothers living with HIV in their country of residence and the socio-cultural factors that influenced their infant feeding practices (i.e., EFF, mixed feeding, EBF).

### Sampling, data collection and management

We surveyed Black mothers living with HIV (*n* = 690) who had at least one child after HIV diagnosis from Port Harcourt (*n* = 400), Miami (*n* = 201), and Ottawa (*n* = 89). Because the target population were hard to reach due to the difficulty of self-identifying as a mother living with HIV, a venue-based sampling strategy was used to select study participants. Venues included churches and mosques, community resource centers, public health facilities, AIDS service organizations, immigrant support agencies, pre-schools, physician’s offices, family gatherings, and other community events. Recruitment was conducted through community intermediaries that introduced the study and gained consent for subsequent contact by a study team member.

Participants completed a demographic questionnaire and instruments to assess functional social support, dimensions of motherhood, and infant feeding attitudes. Data collection occurred between November, 2016 and March, 2018. The prolonged data collection period characterizes the difficulty of reaching the study participants. The survey was conducted by trained medical research personnel and assistants using Qualtrics^XM^ software [13]. In Port Harcourt, participant data was collected using Qualtrics^XM^ software. Data from Port Harcourt was directly entered into Qualtrics^XM^ and was downloaded into SPSS for data cleaning and analysis. In Miami and Ottawa paper-and-pencil surveys were used to collect data that was double entered into SPSS to ensure accuracy of data for analysis. After all data from all three sites were entered into SPSS, they were cleaned and merged into a single aggregate file for analysis. Data entry, cleaning, coding and analyses commenced in 2018.

### Statistical analysis procedures

Sociodemographic characteristics are summarized in Table 1. Functional social support (FSS) was measured using 7-items patterned after the Duke-UNC Functional Social Support Questionnaire [14]. The original instrument included 8-items measured using a visual analog scale with anchors of “much less than I would like” and “as much as I would like.” The instrument was adapted to the specific context of mothers living with HIV and included the following questions: I have people who care about what happens to me; I have chances to talk to someone I trust about my health; I have chances to talk to someone I trust about challenges I face; I have chances to talk to someone I trust about challenges I face with feeding my baby; I get invited to go out and do things with other people including mothers living with HIV; I get useful advice about things that are important to me; I get help when I am sick in bed. Responses were based on a 5-point Likert-type scale of; much less than I would like = 1, less than I would like = 2, some but would like more = 3, almost as much as I would like = 4, as much as I would like = 5. Responses were summed to create a continuous scale ranging from 7 to 35; higher values represent more functional social support. Scale reliability based on Cronbach’s alpha was 0.896.

**Table 1:** Socio-demographic characteristics of Black mothers living with HIV (*N* = 690)

Characteristic	Port Harcourt (*N* = 400)	Miami (*N* = 201)	Ottawa (*N* = 89)	All Sites (*N* = 690)
Age (*μ* ± SD)	34.7 ± 5.7	32.4 ± 5.8	36.6 ± 6.4	34.3 ± 5.9
Years since HIV diagnosis (*μ* ± SD)	6.3 ± 3.5	11.4 ± 7.2	12.7 ± 6.4	8.1 ± 5.6
Persons in household (Mdn; IQR; range)	4; 2; 1–11	3; 2; 1–9	4; 3; 1–6	4; 2; 1–11
Number of children (Mdn; IQR; range)	2; 2; 1–8	2; 2; 1–4	2; 2; 1–5	2; 2; 1–8
Number of children born after HIV diagnosis (Mdn; IQR; range)	1; 1; 1–5	1; 1; 1–3	2; 1; 1–3	1; 1; 1–5
Mothers on HIV treatment: *n* (%)	399 (99.8)	187 (97.4)	50 (96.2)	636 (98.8)
Married: *n* (%)	340 (85.2)	121 (60.8)	29 (33.3)	490 (71.5)
Completed at least secondary education: *n* (%)	352 (89.3)	196 (98.5)	84 (95.5)	632 (92.8)
Employed full or part time: *n* (%)	321 (88.2)	70 (35.2)	60 (67.4)	451 (69.2)

*μ* Mean, *IQR* Interquartile range, *Mdn* Median, *SD* Standard deviation

Multivariate inferential statistics were used to characterize relationships between study variables. Hierarchical binary logistic regression modeling (HBLM) was used to estimate associations between participants’ awareness of infant feeding guidelines and familial, health worker and socio-cultural influences on their infant feeding choices and practices. All tests of significance used the alpha criterion of .05. The outcome variable of interest in the HBLM was participants’ awareness of the infant feeding guidelines in the country where they lived. Participants were asked, “which is the correct national policy on how you should feed your child in the first year of life when you are HIV positive?” For analysis, a mother was considered aware of the guideline if they correctly identified EFF for participants from Canada and the United States and EBF for participants from Nigeria. Responses were dichotomized so practices consistent with guideline adherence were coded 1 and practices inconsistent with guideline adherence were coded 0 (Table 2). Sociodemographic variables (i.e. age, education, marital status, country of residence) were controlled for in this model.

**Table 2:** HIV status disclosure and awareness of national infant feeding guidelines and vertical transmission of HIV among Black mothers living with HIV (*N* = 690)

Indicator	Port Harcourt (*N* = 400) *N* (%)	Miami (*N* = 201) *N* (%)	Ottawa (*N* = 89) *N* (%)	All Sites (*N* = 690) *N* (%)
HIV status disclosure				
Spouse/partner aware of respondents’ HIV status	397 (99.5)	132 (67.0)	81 (92.0)	610 (92.1)
Family aware of respondents’ HIV status	272 (68.0)	149 (80.5)	35 (39.8)	456 (67.2)
Received information about national infant feeding policy from health worker	334 (93.3)	116 (67.4)	50 (57.5)	500 (72.5)
Rated health workers opinion on infant feeding choices as important	393 (99.0)	170 (93.4)	89 (100.0)	652 (97.6)
Aware of national infant feeding guideline for mothers living with HIV	362 (90.5)	144 (72.4)	78 (87.6)	584 (85)
HIV transmission awareness				
Aware HIV can be transmitted from mother to child during pregnancy	352 (88.0)	136 (74.3)	54 (61.4)	542 (80.8)
Aware HIV can be transmitted from mother to child during delivery	355 (88.8)	130 (67.7)	63 (72.4)	548 (80.7)
Aware HIV can be transmitted from mother to child during breastfeeding	353 (88.3)	156 (80.4)	63 (72.4)	572 (84.0)

Predictor variables entered into the HBLM assessed self-perceptions of sociocultural constructs among participants that included: sociodemographic characteristics (Table 1); to whom participants had disclosed their HIV status; and the influence of cultural beliefs or traditions and functional social support (Table 2). Higher functional social support scores indicate that participants perceive more functional social support from within their social networks [15]. Participants were asked whether they perceived that their health workers were aware of the national infant feeding guidelines (Table 2). Prior to entering variables into the final HBLM, analysis of between subjects’ main and interaction effects of each predictor variable on the outcome, guideline awareness, was tested using univariate ANOVA (Table 3). We used Cohen’s *η*^*2*^ effect size categorizations; small = .01, moderate = .059, large = .138 [16]. As a result of these criteria, variables with a statistically significant effect (i.e. cultural beliefs influenced infant feeding choices, functional social support score) and control variables were included in the final HBLM (Table 4). The variable “health worker’s opinion influenced infant feeding choices” was included in the preliminary HBLM. This variable yielded an abnormally large odds ratio (OR = 70.72), indicating near perfect prediction because most participants (*n* = 652, 97.6%) reported health workers influenced their infant feeding choices. The final HBLM model (Table 4) had no evidence of multicollinearity. All predictor variables included in the model had standard errors less than 2. A significant relationship was identified between guideline awareness and the included predictor variables (*Χ*^*2*^ = 38.872, *p* < .05). The reduction in block 2 chi-square error measure, − 2log likelihood (*Χ*^*2*^ *=* 122.967–103.635 = 19.332) was significant (*p* < .01). Hence, socio-cultural variables (block 2) were statistically significant predictors of guideline awareness after controlling for sociodemographic variables (block 1). Among control variables, age was significant (OR = 0.902, *p* = 0.037) with older participants having lower probability of guideline awareness. Interestingly, country of residence as a unique control variable, was not statistically significant (OR = 0.326, *p* = 0.192), making it analytically feasible to pool data from all three countries in one model. Participants who “chose not to answer” survey items were excluded from HBLM analysis.

**Table 3:** GLM univariate ANOVA for variables included in hierarchical binary logistic regression

Outcome variable: awareness of national guideline on infant feeding when living with HIV (aware = 1, otherwise = 0)					
Predictor variable	*df*	MS	*F*	*p*	*η*^*2*^
Country of residence (Canada/USA = 1, Nigeria = 0)	1	.00	.02	.877	.00
Age (years)	27	.02	.68	.865	.20
Formal education (years)	5	.04	1.60	.171	.10
Marital status (married = 1, otherwise = 0)	1	.00	.00	.969	.00
HIV status disclosed to spouse (yes = 1, no = 0)	1	.05	2.06	.156	.03
HIV status disclosed to other family members (yes = 1, no = 0)	1	.05	2.14	.147	.03
Spouse’s opinion influenced IF choices	1	.01	.26	.610	.00
Other family members’ opinion influenced IF choices	1	.00	.00	.952	.00
Health provider’s opinion influenced IF choices	1	.02	.69	.408	.01
**Cultural beliefs influenced IF choices**	**1**	**.29**	**12.13**	**.001**	**.14**
**Functional social support score**	**28**	**.05**	**2.17**	**.004**	**.45**
Country*age	25	.01	.34	.998	.10
Country*marital status	1	.00	.01	.905	.00
Country*formal education	5	.00	.18	.969	.01
Country*HIV disclosure to spouse	1	.00	.03	.872	.00
Country*HIV disclosure to other family members	1	.00	.08	.778	.00
Country*spouse’s opinion influenced IF choices	1	.00	.07	.788	.00
Country*other family members’ opinion influenced IF choices	1	.02	.82	.369	.01
Country*health provider’s opinion influenced IF choices	1	.00	.16	.693	.00
Country*influence of cultural beliefs influenced IF choices	1	.00	.08	.780	.00
Country*functional social support	26	.01	.39	.996	.12
Intercept	1	.55	22.90	.000	.24
Corrected Model	133	.04	1.87	.002	.77

*MS* Mean squares, *η*^*2*^ Effect size, *IF* Infant feeding. Statistically significant (*α* = .05) variables are bolded

*R*^*2*^ = .771 (Adjusted *R*^*2*^ = .359)

**Table 4:** Hierarchical binary logistic regression model of associations between socio-cultural factors and awareness of infant feeding guidelines among Black mothers living with HIV (*n* = 416)

Dependent Variable: Black Mothers’ awareness of the guideline (1 = correct, 0 = wrong)					
Independent variables	EXP(B)	SE	Wald stat	*p*-value	95% C.I. for EXP(B)
*Block 1*					
**Age (years)**	**0.902**	**0.049**	**4.366**	**0.037**	**0.82–0.99**
Years of formal education	0.761	0.169	2.605	0.106	0.55–1.06
Marital status (1 = married, 0 = Single, widowed, divorced or separated)	0.409	0.674	1.759	0.185	0.11–1.53
Country of residence (1 = Canada or US, 0 = Nigeria)	0.326	0.860	1.701	0.192	0.06–1.76
*Block 1 Summary*					
*Χ*^*2*^ = 19.540; Prob(*Χ*^*2*^) = 0.001; −2log likelihood = 122.967					
*Block 2*					
Disclosure of HIV Status to spouse, partner or the baby’s father (1 = yes, 0 = No)	2.072	0.720	1.024	0.312	0.51–8.50
Disclosure of HIV Status to other family members (1 = yes, 0 = No)	0.687	0.387	0.603	0.534	0.21–2.24
Baby’s father’s opinion influenced infant feeding choices (1 = important, 0 = unimportant)	1.246	0.672	0.107	0.744	0.33–4.65
Family member’s opinion influenced infant feeding choices (1 = I care, 0 = I don’t care)	1.478	0.651	0.360	0.549	0.41–5.30
**Cultural beliefs influenced infant feeding choices (1 = much, 0 = not much)**	**0.133**	**0.706**	**8.169**	**0.004**	**0.03–0.53**
**Functional Social Support (scores from psychometric scale)**	**1.100**	**0.045**	**4.496**	**0.034**	**1.01–1.20**
Constant	20,604.34	3.218	9.531	0.002	
*Block 2 Summary*					
*Χ*^*2*^ = 19.332; Prob(*Χ*^*2*^) = 0.004; −2log likelihood = 103.635					
*Overall model Summary*					
*Χ*^*2*^ = 38.872 Prob(*Χ*^*2*^) = 0.000 Sample size included = 416 (60.03%)					
Model accuracy = 95.5%; accuracy of alternative model with outliers = 87.5%.					

Statistically significant (*α* = .05) variables are bolded

## Results

### Demographic characteristics

Table 1 summarizes key demographic characteristics of participants whose average age was 34 years and ranged from 18 to 49 years old. The average number of years since HIV diagnosis was 8.1 years and varied from 6.3 years in Port Harcourt to 12.7 years in Ottawa. Median household size for all sites was four persons and ranged from 1 to 11 persons. The median number of children reported for all sites was two and ranged from 1 to 8, with participants in Port Harcourt reporting the highest number of children. All participants had at least one child after their HIV-positive diagnosis. Participants in Ottawa reported a median of two children born after their HIV diagnosis and participants in Port Harcourt had as many as five children after their HIV diagnosis. Self-reported ART use was high with 98.8% (*n* = 636) of the total sample prescribed ART. Most participants (*n* = 632, 92.8%) had completed secondary education or higher, with participants in Miami reporting the highest education completion level (*n* = 196, 98.5%) and Port Harcourt the lowest (*n* = 352, 89.3%). Participants reported a wide range of employment (full- or part-time), with the highest employment rate in Port Harcourt (*n* = 321, 88.2%) and the lowest in Miami (*n* = 70, 35.2%).

### Awareness of infant feeding guidelines

Awareness of infant feeding guidelines among participants are summarized in Table 2. Overall, awareness of national infant feeding guidelines was high (*n* = 584, 84.6, 95% CI 82.3, 86.8). However, across all study sites, 9.5% (*n* = 38 in Port Harcourt), 27.6% (*n* = 54 in Miami), and 12.4% (*n* = 12 in Ottawa) of participants were **NOT** aware of or “chose not to answer” about their country’s infant feeding guidelines. The majority of participants in Port Harcourt (*n* = 362, 90.5%) were aware that the national guideline on infant feeding when you are HIV-positive is EBF. In contrast, the majority of mothers in Miami (*n* = 144, 72.4%) and Ottawa (*n* = 78, 87.6%) were aware the national guideline prescribed EFF. Most participants (*n* = 500, 72.5%) had received information about the national infant feeding guidelines from their health workers and most (*n* = 509, 73.8%) perceived that their health workers supported the national infant feeding guidelines (Table 2).

### Associations between socio-cultural factors and guideline awareness

The final HBLM revealed that guideline awareness was statistically significantly associated with cultural beliefs and functional social support (*Χ*^*2*^ = 38.872, *p* < .05; Table 4). Participants who reported that cultural beliefs influenced infant feeding choices (OR = 0.133, *p* = .004) were less likely to have guideline awareness. Participants who reported higher functional social support (OR = 1.1, *p* = 0.034) were more likely to have guideline awareness.

## Discussion

It is reassuring that the majority of participants in our study were aware of the infant feeding guidelines for mothers living with HIV (*n* = 584, 84.6%). However, our finding that 15.4% (95% CI 13.2, 17.7) of participants were **NOT** aware of their country’s infant feeding guidelines across our three sites is troubling. These findings are especially troubling because of the increased risk of vertical HIV transmission when mixed feedings are practiced and the potential for legal challenges (e.g., criminal prosecution and/or the loss of their children in Canada and the US) when guidelines are not followed. Additionally, the potential long-term effects of family disruption that these policies can create may be a source of further difficulties for these women, their families and communities. Research is needed to determine how best to increase awareness of infant feeding guidelines among mothers living with HIV and how these guidelines can be reconciled with the social and cultural pressures placed on Black mothers living with HIV “to be good mothers” [12]. Furthermore, there is a need to determine how to reconcile divergent infant feeding guidelines in a globalized world with effective HIV treatments where nuanced differences between WHO and national guidelines in some countries may create perceptions of inequitable approaches to care for mothers living with HIV and their families [8–11].

Most participants in Miami and Ottawa practiced EFF and were adherent with national guidelines, but a high percentage of them were either **NOT** aware of or had misconceptions about the guidelines. In Port Harcourt, most mothers living with HIV were aware of national guidelines on infant feeding. Our finding that most mothers received infant feeding guideline information from their health workers suggests that there may be a disconnect between the national guideline and information passed on by those providers, which may confirm the findings of Tuthill and colleagues [8]. Our findings highlight a need for clearer infant feeding guideline understandings in Port Harcourt and more sensitization efforts in all three study sites. Factors associated with awareness of infant feeding guidelines among Black mothers living with HIV include HIV status disclosure to family members and health workers, which may be essential for mobilizing functional social support. HIV status disclosure was not associated with guideline awareness, most likely because most participants had disclosed to family members and health workers thereby limiting variability in the sample.

### Familial influences on infant feeding guideline awareness

Infant feeding guideline awareness among Black mothers living with HIV has been influenced by the perspectives of their family members, with more than 70% of mothers living with HIV in one study in southern Nigeria reporting family members influenced their decision to practice EBF [17]. In contexts, including the US, where EFF is recommended fathers have been observed administering other types of feedings against the advice of health workers [8]. In our study, it is possible that infants’ fathers may have influenced guideline awareness and the decisions of participants to adhere to the national infant feeding guidelines in Port Harcourt and Ottawa.

### Health worker influences on infant feeding guideline awareness

Health workers are uniquely positioned to influence national infant feeding guideline awareness among mothers living with HIV. Enabling factors such as advice from health workers can influence infant feeding choices, resources available in the community, socio-economic disadvantage (e.g., high cost of formula, limited understanding of EBF), nutritional factors, and mitigate family member pressures for adherence to cultural norms about infant feeding practices [12]. Effective infant feeding counseling may be impeded if healthcare centres are too understaffed to accommodate this specialized public health approach [18]. Our findings provide additional evidence of the importance of health workers to improve guideline awareness among mothers living with HIV as measured by adherence to national infant feeding guidelines [19–21].

Health workers can have divergent and significant influences on mothers’ awareness of infant feeding guidelines. Recent studies have reported that significant numbers of trained health workers have portrayed vertical HIV transmission through breastfeeding as a certainty and not a risk [8, 18], which may result in infant feeding counseling that is inconsistent with approved guidelines. These findings reinforce the need for health workers to be well educated about the rationale and intent of national infant feeding guidelines for mothers living with HIV so they can correctly and effectively communicate with their patients.

### Functional social support, cultural beliefs and infant feeding guideline awareness

Maternal awareness of infant feeding guidelines and understanding of vertical HIV transmission; existential perceptions including values or beliefs that can explain infant feeding choices within the African culture; and negative perceptions that contribute to fears about vertical HIV transmission have been documented to influence infant feeding practices among Black mothers living with HIV [12]. This study found significant but negative associations between cultural beliefs and awareness of correct national infant feeding guideline recommendations for Black mothers living with HIV. This finding is consistent with existing literature suggesting that most Black African cultures do not support recommendations of either EBF or EFF, although breastfeeding is the cultural norm [18, 20].

Furthermore, strong associations between health seeking behaviours associated with awareness of policies and cultural beliefs have been documented [18, 22]. Cultural belief is a dynamic force that often influences responses to treatment and subsequent outcomes [23]. Culturally responsive health counseling can increase counselor credibility as well as among patients, increase satisfaction, self-disclosure, and greater willingness to continue in care [18, 24–27]. This suggests that adherence to infant feeding guidelines among mothers living with HIV can be effectively communicated and inculcated when the policy in itself is culturally adapted and the mode of communication is culturally acceptable.

Limitations of this study include its cross-sectional design, which limits our ability to understand the effects of public health policies and infant feeding guidelines over time. The self-report nature of the survey limits our ability to describe the perspectives or intentions of family members and health workers in relation to infant feeding guidelines. This limits our analysis to participants’ perceptions about the intentions and perspectives of their family members and health workers. Future studies should seek to understand the intentions and perspectives of family members and health workers when they influence the infant feeding choices of Black mothers living with HIV. Additionally, we were limited to self-report data related to ART and its efficacy and did not determine whether mothers had achieved undetectable viral load levels. However, the high percentage of participants who reported being prescribed ART is a hopeful sign because they are engaged in care, which increases the likelihood of undetectable viral load.

### Policy implications

This study may contribute to the development of socio-culturally acceptable evidence-informed infant feeding guidelines for mothers living with HIV and their infants. Strengthening messages about infant feeding options during antenatal care through infant feeding counseling and increasing the awareness of the importance of guideline adherence, whether EFF or EBF, among mothers living with HIV holds promise for improving adherence to infant feeding guidelines as well as the health and wellbeing of mothers living with HIV and their infants.

## Conclusions

Social, cultural, and emotional factors influence HIV-positive mothers’ awareness of infant feeding guidelines and may influence their infant feeding choices. Consequently, there may be inadequate consideration of these factors in the development of infant feeding guidelines. Family members and health workers have the potential to influence infant feeding guideline awareness among mothers living with HIV. Therefore, culturally adapted messaging via social supports already identified by mothers, including family relationships and health workers, is an appropriate way to enhance guideline awareness among mothers living with HIV. Ultimately, contributing to the global health goals of maternal health and reduced infant mortality.

## Data Availability

Primary data analysis activities are currently underway. To request access to study data, please contact JE, the principal investigator for the project.

## Abbreviations

ART: Antiretroviral therapy
EBF: Exclusive breastfeeding
EFF: Exclusive formula feeding
HBLM: Hierarchical binary logistic regression model (ing)
HIV: Human Immunodeficiency Virus
WHO: World Health Organization
